# Organoids and Their Use in Modeling Gut Epithelial Cell Lineage Differentiation and Barrier Properties During Intestinal Diseases

**DOI:** 10.3389/fcell.2021.732137

**Published:** 2021-08-13

**Authors:** Dianne Pupo Gómez, Francois Boudreau

**Affiliations:** Department of Immunology and Cell Biology, Université de Sherbrooke, Sherbrooke, QC, Canada

**Keywords:** intestinal epithelial cells, gut organoids, epithelial barrier, inflammatory bowel disease, inducible pluripotent stem cells, embryonic stem cells

## Abstract

Maintenance of intestinal epithelium homeostasis is a complex process because of the multicellular and molecular composition of the gastrointestinal wall and the involvement of surrounding interactive signals. The complex nature of this intestinal barrier system poses challenges in the detailed mechanistic understanding of intestinal morphogenesis and the onset of several gut pathologies, including intestinal inflammatory disorders, food allergies, and cancer. For several years, the gut scientific community has explored different alternatives in research involving animals and *in vitro* models consisting of cultured monolayers derived from the immortalized or cancerous origin cell lines. The recent ability to recapitulate intestinal epithelial dynamics from mini-gut cultures has proven to be a promising step in the field of scientific research and biomedicine. The organoids can be grown as two- or three-dimensional structures, and are derived from adult or pluripotent stem cells that ultimately establish an intestinal epithelium that is composed of all differentiated cell types present in the normal epithelium. In this review, we summarize the different origins and recent use of organoids in modeling intestinal epithelial differentiation and barrier properties.

## Introduction

**FIGURE 1:**
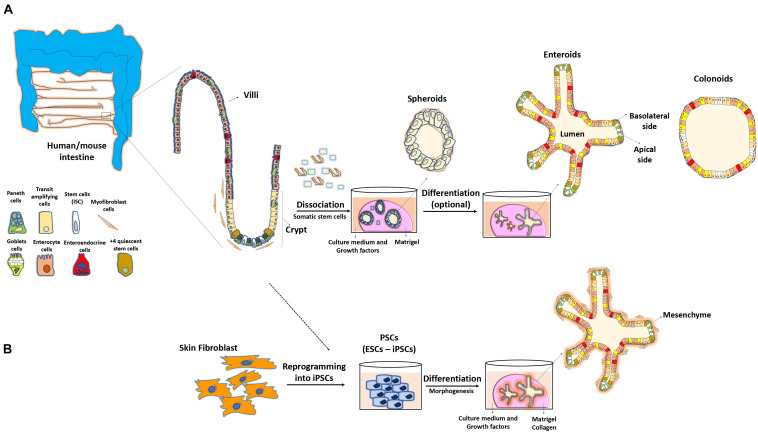
Methodology to obtain 3D intestinal organoids. **(A)** Adult stem cells from isolated intestinal crypts allow the development of three-dimensional cultures reproducing the structure and physiology of the intestine. These mini-guts are embedded in a semi-solid medium (Matrigel) and are grown in the presence of different factors sustaining stability of their niche, cellular differentiation and maintenance in culture for long periods of time. **(B)** Pluripotent stem cells (PSCs) or reprograming of skin fibroblasts can also lead to the formation of these 3D systems.

The gastrointestinal tract (GI) is the main organ related to digestive functions, including absorption and transport of nutrients, water, and electrolytes, and secretion of proteins into the intestinal lumen for maintaining the balance or homeostasis. The intestinal epithelium is an effective barrier against the invasion of microorganisms or infectious agents (Turner, 2009). The intestinal epithelium is composed of a wide layer of specialized and polarized cells interconnected via their membranes including basement membrane through protein complexes (Clevers, 2013). The multipotent intestinal stem cells (ISCs) located at the crypt base generate transit-amplifying cells, which divide successively to generate the following six main types of well-differentiated intestinal epithelial cells (IECs): goblet cells, Paneth cells, enteroendocrine cells, absorptive cells, tuft cells, and microfold (M) cells. These cells migrate toward the tip of the villus or the apex of the crypt in the colon and perform specific functions in the epithelium (Figure 1A). Paneth cells are located at the bottom of the crypt and are involved in epithelial defense as well as stem cell maintenance (Gassler, 2017). Goblet cells are responsible for the synthesis and secretion of mucus, whereas enteroendocrine cells produce hormones and neuropeptides that differ along the rostro-caudal axis of the GI tract. Absorptive cells are involved in metabolic and digestive functions, as well as in the generation of innate immune response due to the expression of specific receptors on their surface (Van Der Flier and Clevers, 2009; Pott and Hornef, 2012). Tuft cells exhibit activity against helminths, and M cells are associated with immunological vigilance and maturation via the recognition of luminal antigens or microorganisms and their subsequent presentation to the underlying immune cells (Peterson and Artis, 2014).

The main functions of the epithelial barrier are maintaining the balance of ions, nutrients, and water passage from the lumen to the organism and restricting the translocation of luminal antigens such as microorganisms and their harmful derivatives. The imbalance in intercellular junctional organization and loss of the intestinal barrier function can lead to the onset of various diseases, including inflammatory bowel diseases (IBD). The integrity of the intestinal epithelial barrier is important in IBD, as it constitutes the delimiting factor for the exposure of microbiota to the host immune system. Consequently, defects in intestinal mucosa homeostasis can trigger alterations in intestinal permeability. This leads to antigen translocation and signaling cascade activation causing apoptosis, erosion, and ulceration, which are considered crucial steps in the initiation or development of chronic intestinal inflammatory disorders (Zeissig et al., 2004; Schulzke et al., 2006; Mankertz and Schulzke, 2007; Antoni et al., 2014).

Defects in the mucus layer can also trigger IBD manifestation. Polymorphisms in some mucin (*MUC)* genes are associated with IBD pathogenesis (Kyo et al., 2001; Moehle et al., 2006; McCole, 2014). Loss of functional MUC2 led to spontaneous manifestation of colitis in mice (Van der Sluis et al., 2006; Heazlewood et al., 2008). Interestingly, Visschedijk et al. (2016) observed the development of ulcerative colitis (UC) in patients carrying MUC2 rare variants. The integrity of the mucus barrier is important in the protection and repair of the epithelium during inflammation. However, whether such defects cause dysregulation in immune system during IBD initiation is unclear. Additionally, factors such as host’s microbiota and immune system can affect intestinal homeostasis. Accordingly, the commensal microbiota plays a determining role in epithelial differentiation (Rakoff-Nahoum et al., 2015). Intestinal physiology also depends on luminal factors including antigens in the diet such as fibers, amino acids, and proteins. Several nutrients come in contact with microbiota and epithelium, and are an essential stimulus in the control and development of the epithelial barrier (De La Serre et al., 2010; Everard et al., 2013; Anhê et al., 2015; Lerner and Matthias, 2015; Gil-Cardoso et al., 2016).

Because of the cellular and molecular complexity of the intestine, elucidating the overall intrinsic mechanisms underlying the regulation of epithelial homeostasis is difficult. In this regard, the use of cell lines has been beneficial (Ponce de León-Rodríguez et al., 2019), however, the immortalized or cancerous nature of such models have limitations in mimicking the normal cellular gut epithelium composition. The recent developments in three-dimensional gut organoid culture systems have revolutionized the basic and biomedical science research. These systems are characterized by high regeneration potential from both normal and diseased primary tissues, and are mini-guts mimicking the physiological features of their tissue of origin. Various methodologies based on the use of whole intestinal crypts that contain adult somatic stem cells or the use of pluripotent stem cells, either embryonic stem cells (ESCs) or induced pluripotent stem cells (iPSCs), have been optimized to allow the formation of villus-crypt-like structures capable of long-term self-renewal (Sato et al., 2009, 2011; Jung et al., 2011; Múnera and Wells, 2017).

## Cell Sources for Organoid Production and Targeted Modeling

### Organoids Derived From Intestinal Crypts

Gut organoid culture was first described using mouse small intestinal segments (Sato et al., 2009). This approach was further expanded to other portions of the intestine and was referred to as “enteroid” culture when the small intestinal origin was used or “colonoids” when the colon was used as a source (Stelzner et al., 2012). One crucial cellular component driving the formation of enteroids is the Lgr5+ stem cell lineage, which leads to the production of polarized and differentiated enterocytes, goblet, enteroendocrine, and Paneth cells (Sato et al., 2009). Lgr5+ cells form a layer that initially resembles a spheroid with subsequent formation of invaginations, simulating the fission of the crypts and intestinal architecture observed *in vivo* (Sato and Clevers, 2013). A study reported that Paneth cells are crucial for the generation of signals essential for the maintenance of ISCs and organoids (Sato et al., 2011). Even though these so-called mini-guts were found to be able to grow *in vitro* without a need of mesenchymal niche, the minimal growth conditions for these entities include factors and extracellular molecules that normally compose this niche. More precisely, Matrigel, a semi-viscous medium enriched with the extracellular matrix, provides the essential microenvironment for IEC self-renewal, differentiation, and cell–cell interactions (Figure 1A). In addition, a cocktail of biological enhancers such as the bone morphogenetic protein inhibitor Noggin, epidermal growth factor, R-spondin-1, and Wnt3a is required for IEC expansion and maintenance under the culture conditions (Ootani et al., 2009; Sato et al., 2009, 2011). An additional conserved feature of organoids compared with the *in vivo* system is that organoids exhibit the luminal region in which apoptotic enterocytes and metabolites are expelled. However, in contrast to the gut mucosa, where the external milieu is in contact with the enterocyte apical side, the polarized apical side of enterocytes faces to the inside of organoids, whereas the basolateral region is in contact with the growth medium (Sato et al., 2009). A recent strategy has been optimized to reverse this polarity by eversion of the apical side facing the culture medium (Co et al., 2019).

The use of the CreER/LoxP system, which allows conditional deletion of a targeted gene in an inducible manner, is a powerful tool for determining the role of various molecules in gut organoids that mimic intestinal pathologies (Bohin et al., 2018; Montenegro-Miranda et al., 2020). The recent establishment of organoids derived from the biopsies of patients with intestinal diseases provides the utmost advantage of exhibiting the similar characteristics of the primary diseased tissue from the perspective of developing personalized therapies. d’Aldebert et al. established and characterized colonoids from patients with IBD and compared them with those of healthy individuals. They concluded that despite these colonoids exhibit a similar cellular composition during the first 12 days of culture, IBD-derived organoids exhibit the characteristics of inflammation with increased cell death, reversal of cell polarization, and decreased expression of tight junction proteins (d’Aldebert et al., 2020). Noben et al. reported that IBD-derived colonoids are representative tools for assessing the molecular alterations responsible for the different types of inflammatory disorders in these patients. For example, the organoid cultures derived from patients with Crohn’s disease (CD) had a remarkable decrease in the expression of *MUC2* gene transcripts as determined by qPCR and when compared with the organoids derived from healthy individuals or patients with UC. However, the inflammatory gene transcript signatures were not maintained compared with the original biopsies or the primary tissue sites of the corresponding patients (Noben et al., 2017).

### Organoids and Epithelial Barrier Properties

**FIGURE 2:**
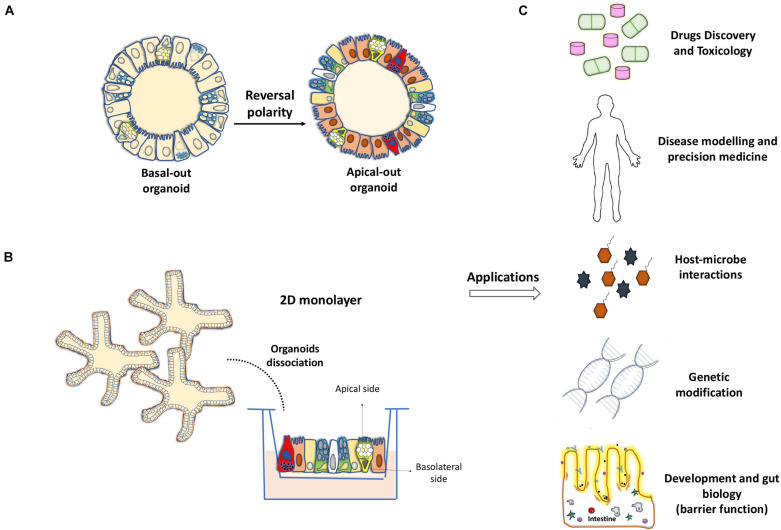
Development of 2D monolayers and organoids with reverse polarity for their application in the study of intestinal biology. **(A)** Reversal of the cell polarity of intestinal organoids in the absence of extracellular matrix proteins, allows direct access to the apical surface of the epithelium and facilitates interaction with microbes and other molecules. **(B)** Transwell-based 2D monolayer culture of IECs can be obtained from the dissociation of enteroids or colonoids. **(C)** Applications for organoid models such as drug development, genetic manipulation or pathogen-host interactions and their key uses for basic science and precision medicine.

Several reports have used gut organoids in studies pertaining to epithelial barrier functions and host–pathogen interactions. Using enteroids from wild-type C57BL/6 mice, Farin et al. (2014) observed a high and rapid sensitivity of Paneth cells toward degranulation and release of microbial peptides in the presence of interferon-γ, without generation of a similar response toward various molecular patterns associated with the microbes. The aforementioned apical-out enteroids not only retain their capacity for cell differentiation and native intestinal functions but also are suitable for barrier permeability assays including 4 kDa fluorescein isothiocyanate-dextrans (Co et al., 2019; Figure 2A). Other experimental protocols include dissociation of organoids, allowing the formation of polarized 2D monolayers directly on the pore membranes of Transwell systems (Kozuka et al., 2017; Tong et al., 2018; In et al., 2019; Figure 2B). Significant efforts are needed to obtain and reproduce the monolayers that mimic different properties of the tissue of origin, such as *in vivo* epithelial cell organization, epithelial cell absorption and transport mechanisms, and transepithelial electrical resistance (Fernando et al., 2017; Altay et al., 2019). Recently, Wang et al. showed that the fragmented colonic spheroids seeded as single cells on Transwells and exposed to an air–liquid interface for 21 days can recapitulate morphological changes and patterns of cell differentiation. This allowed goblet cells to produce a thick protective mucus layer covering the epithelium, opening the new possibilities for investigating the intestinal barrier under these conditions (Wang et al., 2019). On the other hand, Moon et al. (2014) used culture monolayers from colonoids of multiple genetic mouse strains to study the role of polymeric Ig receptor during immunoglobulin A transcytosis. Epithelial barrier dysfunction in the context of pathogen exposure has also been studied (Noel et al., 2017; Nakamura, 2019). A group of researchers established suitable enteroid monolayers derived from the human fetal small intestine to study viral replication of Enterovirus A71 and bacterial translocation of *Listeria monocytogenes* after apical infection (Roodsant et al., 2020). The colonoids generated from patients with CD could reproduce epithelial barrier properties, as observed in the native intestinal epithelium, based on the expression profile of key junctional components (ZO-1, OCLN, CLDN4, and CTNNB) and paracellular permeability assays using fluorescein isothiocyanate-dextran (Xu et al., 2018).

### Organoids and IEC Lineage Differentiation

Gut organoid culture has provided important clues as to how IECs can integrate external signals from the environment and intrinsically affect cell fate determination during epithelial renewal. The high Wnt activity was found to be central to IEC determination. The high expression of Wnts favors ISC proliferative status and commitment of Paneth cells, whereas the low expression leads to the differentiation of enterocytes and goblet cells (Farin et al., 2014, 2016; Tian et al., 2015; Kim et al., 2020). Thus, pharmacological activators or inhibitors of Wnt, in addition to other pathways such as Notch and ROCK, can be used to manipulate epithelial cell differentiation in the small intestine toward a specific cellular fate (Yin et al., 2014; Beumer et al., 2018; Petersen et al., 2018). Similar strategies have been optimized recently for human and mouse colonoids (Wilson et al., 2021). Another study has reported the reprograming capacity of a specific cocktail of transcriptional factors (HNF4γ, GATA6, CDX2, and FOXA3) to produce gut-like organoids from mouse fibroblasts (Miura and Suzuki, 2017). HNF4γ was also proposed to act as a major driver of enterocyte differentiation by coupling the use of mouse enteroids with integrative systems biology analysis (Lindeboom et al., 2018). Interestingly, Cldn7 depletion in mouse enteroids revealed the crucial role for this tight junction membrane protein in the regulation of ISC survival as well as the differentiation of IEC cell lineage differentiation (Xing et al., 2020). Organoids offer thus exciting opportunities to elicit differentiation- related mechanisms in the context of developmental biology and medicine (Figure 2C).

### Organoids Derived From Isolated ESCs and iPSCs

The organoids derived from ESCs and iPSCs have emerged as an alternative approach when gut tissues are somewhat limited, or when invasive procedures are simply not possible for human patients. This method relies on specifying the progenitor cells from iPSCs with an optimized protocol sequence involving specific signals, factors, and culture conditions to ultimately stimulate their differentiation and subsequent formation of *ex vivo* organoids (Forbester et al., 2015; Dotti et al., 2017; Hibiya et al., 2017). Unlike other cellular systems, the organoids produced by this method simultaneously promote the presence of mesenchymal cells (Figure 1B), that surround the intestinal epithelium, forming a cellular microenvironment similar to that observed in the intestine (Spence et al., 2011; Gjorevski et al., 2016; Takahashi et al., 2018). Although many single organoids can regenerate from iPSCs, these structures do not recapitulate the mature differentiation process, and rather show characteristics similar to the embryonic development of the epithelium, which limits their use in the functional analysis of gut adult stages (Spence et al., 2011; Fordham et al., 2013). Furthermore, evidence supports that the organoids derived from PSCs exhibit a high tendency for tumor formation in the absence of specific, controlled growth conditions (He et al., 2020). Nevertheless, considerable efforts have been made to improve this method and to bring these models closer to the physiology related to the mature intestinal tissues. One study enhanced mini-gut maturation and cellular vascularization by generating enteroids from human ESCs or iPSCs that were subsequently engrafted *in vivo* into the kidney capsule of immunocompromised mice (Watson et al., 2014). Another group developed the first model of human intestinal organoids with a functional enteric nervous system using PSC-derived neural crest cells that allowed contractile activity (Workman et al., 2017). Similarly, Holloway et al. (2020) grew and co-differentiated endothelial cell populations *in vitro* within the culture of human PSC-derived intestinal organoids, with characteristics similar to those of the native endothelial cells, and thus ensuring vascularization of this system. Thus, ESCs and iPSCs are powerful tools for the study of normal intestinal development (McCauley and Wells, 2017; Perez-Lanzon et al., 2018; Sugimoto et al., 2018; Fowler et al., 2020) or for elucidating the mechanisms underlying the onset of diseases such as cancer (Crespo et al., 2017; Smith and Tabar, 2019). However, an important limitation to consider while designing such experimental strategies is the genetic heterogeneity of iPSCs, as opposed to crypt-derived IECs, that can further affect the phenotypes and gene expression of differentiated cells under these assays (DeBoever et al., 2017; Kilpinen et al., 2017).

### Challenges and Future Perspectives

Organoids are 3D structures, which have become the potential tools for the study of *ex vivo* intestinal physiology. While mouse organoid experiments can be functionally validated *in vivo* with available genetically modified mouse models, human organoids provide new perspectives into human biology. However, the expansion of progenitor cells that constitute the fundamental basis of this constantly-renewing system faces challenges in terms of reproducibility among different laboratories. The variations involved in this process range from individual manipulation in the laboratory; oxygen levels and the source and quality of growth medium factors used in the cultures; methodologies of stem cell isolation from the primary tissues; and genetic and epigenetic variability of the cellular source (Shuhendler et al., 2013; Lehmann et al., 2019). All these parameters represent important caveats for the successful establishment of gut organoid cultures. Another important aspect while studying the characteristics of these cultures is the absence of various cell types that normally interact with epithelial cells to maintain the homeostasis in intestinal epithelium. These include immune, mesenchymal, endothelial, muscle, and nerve cells, as well as microbes and their metabolites. Setting up mini-gut co-culture conditions with different cell types, such as isolated intraepithelial lymphocytes, monocytes/macrophages/neutrophils, or fibroblasts are attracting the possibility of reproducing the *in vivo* intestinal microenvironments under these conditions (Nozaki et al., 2016; Pastuła et al., 2016; Staab et al., 2020). In addition, the recent advances offer new possibilities for the genetic engineering of organoid culture systems. CRISPR/Cas9 technology is one of the most commonly used genomic editing techniques that combines Cas9 endonuclease action to short guide RNA sequences specifically binding to genomic target sequences (Drost et al., 2017; O’Rourke et al., 2017; Roper et al., 2018). The recent inclusion of bioengineering strategies to build biomimetic-based or microfluidic-based scaffolds, gut organoid-on-a-chip which are built as microfluidic devices allowing continuous perfusion as it is the case in a living tissue, and organoid-derived intestinal grafts (Rahmani et al., 2019) might enhance our understanding of the overall structural and cellular complexity of organoid culture.

## Conclusion

Organoid culture has emerged as an important biological tool for the study of intestinal epithelial biology. Understanding the mechanisms underlying the successful establishment of mini-gut from stem cells and mechanisms underlying the IEC-differentiation process can provide new knowledge for developing accurate organoid models. These models will be able to reproduce with greater fidelity for the intrinsic epithelial pathological defects that affect the intestine during intestinal diseases. The culture of gut organoids has been instrumental in testing the functional integrity of the barrier normally established between the host and possible surrounding pathogens. This combined with the recent development of new genetic technologies can provide potential opportunities for implementing this *ex vivo* culture system in the basic and clinical research for the purpose of regenerative medicine and personalized therapies (Figure 2C).

## Author Contributions

DG and FB wrote the manuscript. FB provided the funding. Both authors contributed to the article and approved the submitted version.

## Conflict of Interest

The authors declare that the research was conducted in the absence of any commercial or financial relationships that could be construed as a potential conflict of interest.

## Publisher’s Note

All claims expressed in this article are solely those of the authors and do not necessarily represent those of their affiliated organizations, or those of the publisher, the editors and the reviewers. Any product that may be evaluated in this article, or claim that may be made by its manufacturer, is not guaranteed or endorsed by the publisher.

## Abbreviations

GI: gastrointestinal tract
ISCs: intestinal stem cells
IECs: intestinal epithelial cells
IBD: inflammatory bowel diseases
UC: ulcerative colitis
CD: Crohn’s disease
*MUC*: mucin genes
ESCs: embryonic stem cells
iPSCs: induced pluripotent stem cells.
